# Determinants of Acute Malnutrition among Under-Five Children in Governmental Health Facilities in Sodo Town, Southern Ethiopia: Unmatched Case-Control Study

**DOI:** 10.1155/2023/3882801

**Published:** 2023-07-18

**Authors:** Zenebe Jebero, Fikre Moga, Bereket Gebremichael, Tewodros Tesfaye

**Affiliations:** ^1^College of Medicine and Health Sciences, Arba Minch University, Arba Minch, Ethiopia; ^2^College of Health Sciences, Addis Ababa University, Addis Ababa, Ethiopia

## Abstract

**Background:**

Acute malnutrition is a major public health challenge among children globally. The burden is high in low-income countries like Ethiopia. Different reports and literatures revealed different risk factors of acute malnutrition in different geographical areas, but there were regional variations. So, the main aim of this study was to identify determinants of acute malnutrition among under-five children in governmental health facilities of Sodo town, Southern Ethiopia.

**Methods:**

An institutional-based unmatched case-control study was conducted from February 1 to March 1, 2021. Consecutive sampling was used to select cases, and controls were selected using a systematic random sampling technique. An interviewer-administered structured questionnaire was used to collect data, and standardized anthropocentric measurement equipment was used to identify cases and controls. Data were analyzed using SPSS version 26. A logistic regression model was used to identify the determinants of acute malnutrition, and statistical significance was declared at *P* < 0.05.

**Result:**

A total of 133 cases and 266 controls were included in the study making a response rate of 97.8%. Mothers with no formal education, birth interval less than 24 months, marital status (divorced widowed and separated), diarrhea in the past two weeks, using nonprotected water for drinking, exclusive breastfeeding less than 6 months, not taking sick children to health facility within 24 hours of the onset of any sickness, low birth weight, breastfeeding for less than 24 months, using nonimproved toilet, low dietary diversity, and food insecurity were significantly associated with acute malnutrition.

**Conclusion:**

This study identified the major determinants of acute malnutrition among under-five children in the study area. Thus, ensuring safe water supply, empowering women, and improving knowledge and practices of mothers regarding exclusive breastfeeding and family planning are recommended.

## 1. Introduction

Malnutrition in all forms is defined as deficiencies, excess, and imbalance of a person's intake of energy and important nutrients with body demand [1]. Acute malnutrition is a devastating global human disaster which is considered as both medical and social problems that affects all age group in general and children in particular [2]. Each year, millions of children die because of acute malnutrition. Also, the majority of deaths among under-five children are directly linked to acute malnutrition. Acute malnutrition puts children at greater risk of death which affects individuals, families, communities, and countries as a whole [1, 3, 4].

Globally, one among three children is not growing well due to malnutrition, and in South Asia, even one child among two is malnourished. In South and East Africa, two among five children are undernourished. Despite the alarming magnitude of acute malnutrition in the world particularly in developing countries like Ethiopia, only about one child in four receives treatment of acute malnutrition [5]. In addition, around 28% of child death in Ethiopia is directly associated with malnutrition which is devastating [6].

According to Ethiopian Demographic and Health Surveillance (EDHS) data, ten percent of children were wasted (too thin for their height) and three percent of them were severely wasted. In 2019, 7% and 1% of children are moderately and severely wasted, respectively. The average annual prevalence and trends of acute malnutrition among children are high in Ethiopia particularly among under-five children, and the majority of deaths occur among them. Still, child malnutrition remains a serious public health problem in Ethiopia, and it needs strong and immediate remedial interventions and studies [6–9].

It is well known that acute malnutrition is Ethiopia's main health problem in general and in the study area in particular. Numerous studies and reports have identified a variety of factors that increase the risk and impact of acute malnutrition, including maternal education, poor socioeconomic status, living in rural areas, inadequate dietary intake, diarrhea, inappropriate feeding practices, lack of access to health services, and birth interval [10–13]. However, there are regional differences in the burden and risk factors that contribute to acute malnutrition. Furthermore, the majority of studies on the determinants of acute malnutrition were undertaken in pastoralist and nomadic communities, which cannot be considered representative of other populations.

Moreover, most studies done on similar issues in populations with similar features used cross-sectional study designs, which is inadequate to identify independent predictors of acute malnutrition. Additionally, most studies failed to put enough emphasis on factors that are related to the use of maternal and child health services, such as lack antenatal follow-up, postnatal follow-up, lack of counselling on maternal nutrition during pregnancy, lack of regular growth monitoring, and lack of counselling by health care providers on infant and child feeding which may have a significant impact on child malnutrition. Most of all, even though the burden of acute malnutrition is high in the study area [14–16], the factors behind it are not clearly known. Therefore, this study is aimed at filling the aforementioned gaps by assessing the determinants of acute malnutrition among under-five children in governmental health facilities in Sodo town, Wolaita zone, Southern Ethiopia.

## 2. Method and Materials

### 2.1. Study Area, Design, and Period

The study was conducted in Wolaita Sodo town, in Southern Ethiopia which is an administrative town of Wolaita zone. It is located in 327 km south of Addis Ababa, the capital city of Ethiopia, and 152 km away from Hawassa, the regional city of Southern Nations, Nationalities, and People's Region (SNNPR). In the town, there is one teaching and referral hospital, one private hospital, 3 health centers, and 20 private clinics. This institutional-based unmatched case-control study was conducted from February 1 to March 1, 2021, in governmental health facilities of Wolaita Sodo town, Southern Ethiopia.

### 2.2. Population

#### 2.2.1. Source Population

The source population of this study was all children aged 6-59 months who receive health service in governmental health facilities in Wolaita Sodo town.

#### 2.2.2. Study Population

*(1) For Cases*. All under-five children with weight/height <-2*z*-score of WHO standard and/orMUAC < 12.5with or without bilateral oedema and who visited outpatient or inpatient departments of governmental health facilities in Wolaita Sodo town were selected.

*(2) For Controls*. All under-five children with weight/height >-2*z*-score, MUAC > 12.5, and no bilateral oedema and who visited outpatient or inpatient departments of government health facilities in Wolaita Sodo town for any reason were selected.

### 2.3. Inclusion and Exclusion Criteria

#### 2.3.1. Inclusion Criteria

All children aged 6-59 months, who have acute malnutrition (for cases), and who have no acute malnutrition (for controls) who visited selected health facilities in Wolaita Sodo town during data collection period and those children whose mother/caregiver were present were recruited to the study.

#### 2.3.2. Exclusion Criteria

Children with physical deformities (which will interfere with or give an incorrect measurement), children who were critically ill, and children with congenital anomalies, known chronic diseases like HIV/AIDS, tuberculosis, and chronic heart diseases were excluded from the study.

### 2.4. Sample Size Determination and Technique

The sample size was computed using STAT CALC application of Epi Info version 7 by using the following assumptions: proportion of mothers of controls with no formal education 16.37% and proportions of mothers of cases with no formal education 29.7%; 2.16 OR from the study in East Welega, Oromia region; 5% type I error; 80% power; case to control ratio of 1 : 2; and 10% for nonresponse rate. Thus, the total sample size was 408: 136 cases and 272 counters. All health centers in the town such as Sod town health center, Ganame health center, and Wadu health center and Wolaita Sodo University Teaching Hospital were selected. Then, the calculated sample size was proportionally allocated to the health facilities based on the average monthly flow of the cases from previous months' report. Consecutive sampling was used to select cases, and a systematic random sampling technique was used to select controls.

### 2.5. Data Collection Instruments and Procedures

Data were collected from mother's/caregivers of children by using standardized interviewer-administered questionnaires, and child nutritional status was determined by using anthropometric measurements. Data collection tools were prepared after reviewing different related literatures to address the study objectives. The questionnaire was made up of 74 questions in total, with the first section focusing on the socioeconomic characteristics of the study participants (11 questions), the second section on child characteristics (11 questions), the third section on factors related to the use of maternal and child health services (15 questions), the fourth section on environmental factors (3 questions), and the fifth section on child caring and feeding practices (34 questions). The questions were all closed-ended. The questionnaire was translated into Amharic and local language (Wolaitta language) for fieldwork purpose, and again, it was translated back into the English language by language experts to check its consistency. The weight of the children was taken by using WHO standard instrument and procedures (seca 874 U electronic scale). Daily check of the weighing scale was done to ensure the accuracy of the scale. The check was done by weighing the same object (with a known and constant weight) every morning before fieldwork begins. Children were undressed or worn light clothes, and the weight of the child was measured to the nearest 0.1 kg. For the children who were very small and frightened or upset, the mother's weight was taken first and then weighed while holding the child in her arms, and the scale automatically computed the child's weight by subtraction. The length of children aged below 2 years was measured in the recumbent position by using sliding board, while the standing height was measured for children aged above 2 years, and the height/length of the children was measured to the nearest 0.1 cm. MUAC of the child was also measured on the left arm by using tape meter at the level of upper arm midpoint mark to the nearest 0.1 cm. Also, normal thumb pressure was applied for both feet for three seconds to assess the presence of pitting bilateral oedema. The reliability test indicated that our tool had an adequate internal consistency (Cronbach's alpha = 0.855), and the item content value index of our tool was in acceptable range (Pearson's correlation = 0.71 − 0803).

### 2.6. Operational Definitions

#### 2.6.1. Cases

Case are those whose weight/height was <-2 *z*-score of WHO standard and or MUAC < 12.5 with or without bilateral oedema [17].

#### 2.6.2. Controls

Controls are those under-five children whose weight for height is >-2 and MUAC ≥ 12.5 cm and who have no nutritional oedema.

#### 2.6.3. Food Security

The food security status of the households was determined based on nine standard Household Food Insecurity Access Scale (HFIAS) questions that were developed for this purpose by Food and Nutrition Technical Assistance (FANTA) in 2007. In the HFIAS measurement, each of the questions was asked with a recall period of four weeks (30 days). The respondent was first asked an occurrence question, that is, whether the condition in the question happened at all in the past four weeks (yes or no). If the respondent answers “yes” to an occurrence question, a frequency-of-occurrence question was asked to determine whether the condition happened rarely (once or twice), sometimes (three to ten times), or often (more than ten times) in the past four weeks [18].

#### 2.6.4. Food Secure Households

A household was classified as food secure if respondent did not experience none of the food insecurity questions or just experienced worry rarely (score < 1), otherwise categorized as food insecure [18].

### 2.7. Data Quality Management

To ensure the quality of data, training was given for supervisors and data collectors on the purpose of the study, techniques of data collection, anthropometric measurement techniques, and data recording. Pretest was conducted on 5% of the total sample size outside of the study area. The supervisors and investigator followed the day-to-day data collection process and ensured the completeness and consistency of collected data on daily basis. The anthropometric assessments were done using a standardized technique and equipment; the questionnaire was checked for consistency and completeness.

### 2.8. Data Processing and Analysis

Data were checked for completeness and consistency before data entry, and then, it was entered into EpiData 4.6 and exported to SPSS version 26 for analysis. The data were coded, recoded, and stored to ease up analysis. Logistic regression analysis was done to identify the independent predictors of acute malnutrition. Thus, model fitness was checked. Multicollinearity among independent variables was checked by a variance inflation factor (VIF) and tolerance test. The independently associated variables in bivariate logistic analysis with *P* value < 0.25 were entered into a multivariable logistic regression analysis to control for confounding variables and to identify independent predictors of acute malnutrition. The adjusted odds ratio (AOR) with their respective 95% confidence interval was used, and *P* values < 0.05 were considered as statistically significant.

## 3. Results

### 3.1. Sociodemographic Characteristics

A total of 399 participants (133 (33.3%) cases and 266 (66.7%) controls) were included in the study with an overall response rate of 97.8%. The mean age of mothers of cases and controls was 33.17 and 31.81, respectively. Regarding family size, 54 (40.6%) of cases and 51 (19.2%) of controls had a family size of five and above. About 41.4% of cases and 27.4% of controls were from rural area. Regarding maternal education, high proportion of mothers of cases 37 (27.8%) had no formal education when compared to mothers of controls 14 (5.3%). Besides, individual-based decision-making on money (decision made by either father or mother alone) was relatively higher among cases 80 (60.9%), and the majority of controls 210 (78.9%) make decision jointly (Table 1).

### 3.2. Child Characteristics

Around two-thirds of both cases 76 (57.1%) and controls 166 (62.4%) were males, and the mean age of children was 24.33 months for cases while it was 24.35 months for controls. Concerning the birth weight of the children, 18 (43.9%) and 15 (15.5%) of cases and controls had a birth weight less than 2.5 kg (Table 2).

**Figure 1:**
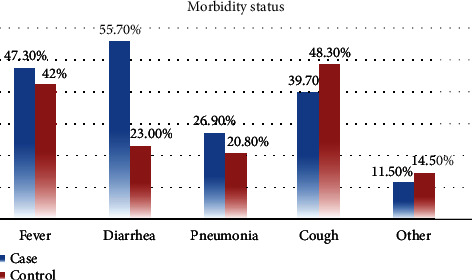
Morbidity status among cases and controls of under-five children, 2021. Note: the *y*-axis in the figure shows the percentage of each illness in the area, while the *x*-axis shows the types of illnesses that are prevalent there. Cases are represented by the blue color and controls by the red color.

Regarding morbidity status, the majority of cases 131 (98.5%) and controls 207 (77.8%) were sick in the past two weeks prior to data collection. Relatively highest proportion of cases had diarrhea in the last two weeks 73 (55.7%) preceding the survey (Figure 1).

### 3.3. Maternal and Child Health Service Utilization

Out of the total study participants, 73 (54.9%) and 191 (71.8%) of mothers of cases and controls had regular ANC visit during pregnancy, and among those who had ANC visit, 52 (71.2%) of cases and 167 (87%) of controls got counselling on diet and nutrition during pregnancy. Regarding place of delivery, 30 (22.6%) of cases and 42 (15.8%) of controls gave birth (delivered) at home. Significant majority of cases 131 (98.5%) and controls 246 (92.5%) had no growth monitoring follow-up in the health facility. Around two-thirds of cases 86 (64.7%) and almost three-fourth of controls 204 (76.7%) have reported to have health facility within 10 km radius of their home. The majority of parents of cases 103 (81.1%) and controls 105 (40.2%) do not take their children to health facilities within 24 hours of onset of sickness.

**Figure 2:**
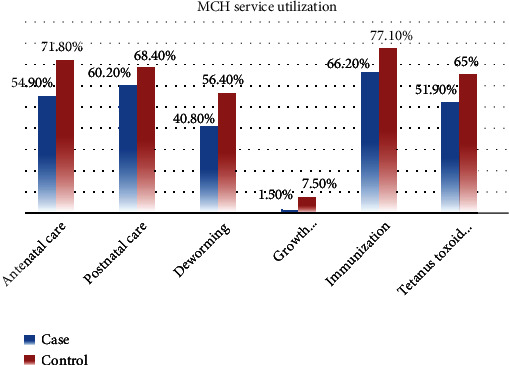
Maternal and child health service utilization among cases and controls under-five children in Sodo town public health facilities, 2021. Note: the *y*-axis shows the percentage of each service that was used, while the *x*-axis shows the different MCH service types. Red color represents controls, and blue color represents cases.

In addition, individual-based decision-making on the care and treatment of the child was 72 (54.1%) among cases while it was 54 (20.4%) among controls (Figure 2).

### 3.4. Environmental Factors

Almost all households (99.2%) of both cases and controls have toilet in their compound, but only 9 (6.8%) and 104 (39.4%) of cases and controls have improved type of toilet, respectively. Concerning drinking water supply, relatively high proportion of households of cases 57 (42.9%) and controls 30 (11.3%) use drinking water from a nonprotected water source.

### 3.5. Child Caring and Feeding Practice

Concerning breastfeeding, the majority of cases 111 (83.5%) and more than one-third of controls 98 (36.8%) had exclusive breastfeeding for less than six months of birth. Around two-thirds of both cases 89 (66.9%) and controls 168 (63.2%) experienced bottle feeding. Besides, the majority 124 (93.2%) of cases and around one-third of controls 97 (36.5%) discontinued breastfeeding before 24 months of their age. Concerning food insecurity, relatively high proportion of cases 117 (88%) were suffering from food insecurity when compared to controls where 124 (46.6%) were suffering from food insecurity. Likewise, around half 62 (46.6%) of cases and one-third 78 (29.3%) of controls did not get counseling on infant feeding practice (Table 3).

**Figure 3:**
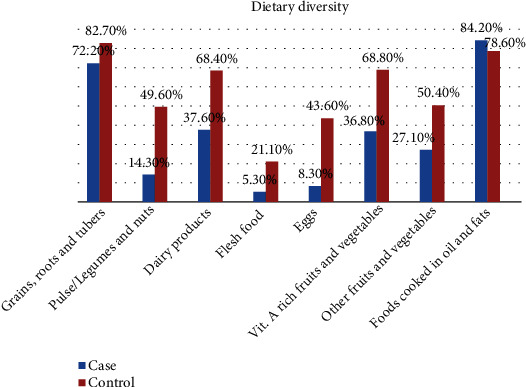
Dietary diversity feeding practice among cases and controls under-five children in Sodo town public health facilities, 2021. Note: the *x*-axis represents the type of diets the children consume in the area, while the percentage of each type of diet is shown on the *y*-axis. The blue color represents cases and red represents controls.

In the case of dietary diversity of the children, most of cases 103 (77.4%) and one-third of controls 88 (33.1%) had inadequate dietary diversity (consumed from less than four groups of foods in the past 24 hours proceeding the interview). Grains were the most commonly consumed category of food by both groups 96 (72.2%) and 220 (82.7%) while flesh foods were rarely consumed by both groups 7 (5.3%) and 56 (21.1%), respectively (Figure 3).

### 3.6. Determinants of Acute Malnutrition

All independent variables were checked for the presence of association with acute malnutrition in bivariate logistic regression analysis. The variables found to be associated with acute malnutrition in bivariate analysis (*P* value < 0.25) were entered into multivariable logistic regression. Multivariable logistic regression analysis showed that the likelihood of being malnourished was higher among children from parents who were not living together/other than married (AOR 4.45 (1.72, 11.51)), family size of five and above (AOR 2.54 (1.37, 4.73)), and birth interval less than 24 months (AOR 2.71 (1.54, 4.76)); children of mothers who have never attended formal education were almost three times more likely to develop acute malnutrition (AOR 2.96 (1.02, 8.56)) and low birth weight (AOR 3.56 (1.15, 10.99)); children who had diarrhea in the past two weeks prior to data collection were around nine times more likely to suffer from acute malnutrition (AOR 9.15 (2.83, 29.53)); and not taking sick children to health facility within 24 hours of the onset of any sickness (AOR 5.10 (2.94, 8.86)), using nonimproved toilet (AOR 5.09 (2.40, 10.79)), using nonprotected water for drinking (AOR 3.45 (2.00, 5.93)), duration of exclusive breastfeeding less than 6 months (AOR 4.65 (2.21, 9.78)), breastfeeding for less than 24 months (AOR 10.26 (4.57, 22.99)), dietary diversity (AOR 2.05 (1.05, 4.00)), and food insecurity (AOR 3.83 (1.80, 8.14)) were significantly associated with acute malnutrition in the study area (Table 4).

## 4. Discussion

This study tried to look into different determinants of acute malnutrition by encompassing various factors. Among the sociodemographic characteristics, children of parents who are not married were 4.45 times more likely to develop acute malnutrition when compared to children whose parents are living together. This finding is consistent with the study conducted in Chad and Karat town [19, 20]. However, this finding is in contrary with the studies done in East Ethiopia and Gambela [21, 22]. The high odds of acute malnutrition among unmarried women in the current study might be due to the high influence of family structure or characteristics on nutritional status and survival of children. In addition, a parent who takes care of children alone might not involve in various economic activities, so they cannot invest in the child's welfare adequately and cannot provide a balanced diet for their children.

In this study, children from parents with a family size of five and above were 2.54 times more likely to suffer from acute malnutrition. This study was consistent with the study conducted in Ethiopia and Nepal [20, 23–25]. But studies from East Gojjam zone [26], Shashogo Woreda [27], and Jimma zone [28] revealed the opposite report. The higher likelihood of acute malnutrition among children from large family size in this study might be because the allocation of food per child is more likely to decrease with the increase in the number of family members in the household, which in turn may adversely affect the nutritional status of the children.

The present study exhibited that nonoptimum birth interval is significantly associated with acute malnutrition. Children with a birth interval of less than 2 years were almost three times more likely to develop acute malnutrition. The studies from different parts of Ethiopia are in agreement with this finding [21, 28]. However, the reports from Afar region [25] and Shashogo Woreda [27] are inconsistent with this finding. This may be because as the birth interval gets short, there may be sharing of breastfeeding among the older and younger children which may end up with the inadequate intake of breast milk by both the old and younger child increasing the risk of being malnourished.

Children from mothers who never attend formal education were three times more likely to develop acute malnutrition when compared to their counterparts. This finding is in agreement with different studies in different regions of Ethiopia and India [22, 24, 27–29]. This could be due to the fact that the more educated mothers have great awareness and knowledge on child caring and feeding practice, and also, they can involve in various economic activity. Therefore, they can provide the most diversified food to their children which decreases the risk of being malnourished.

The present study exhibited that the odds of acute malnutrition among children whose birth weight is less than 2.5 kg is 3.56 times higher when compared to their counterparts. This finding agrees with the study done in India [29] and Chad [19]. This is due to the fact that children whose birth weight is low are prone for infection and other illnesses; this may predispose them to malnutrition.

In the present study, it was found that children who had diarrhea two weeks prior to data collection were around nine times more likely to suffer from acute malnutrition when compared to their counterparts. Similar studies conducted in different parts of Ethiopia [20, 22, 24, 26, 27, 30], Chad, and Kongo [19, 31] support the finding from this study. This is due to the fact that diarrhea causes loss of excessive fluids and important nutrients from the body, reduces appetite, reduces energy intake, causes malabsorption, and also increases the bowel motility of the children which may result in child malnutrition.

This study also showed that acute malnutrition was higher among children from household which uses nonimproved type of toilet and those household drinking water from unprotected source when compared to their counterparts. This result is in agreement with the studies conducted in different parts of Ethiopia [20, 32]. This could be explained by the lack of safe drinking water supply, use of unprotected water, and use of unimproved toilet which are directly related with the incidence of diarrhea and other water- and foodborne diseases which result in child malnutrition.

In the current study, it was found that children who were not taken to health facilities within 24 hours of the onset of any sickness were 5 times more likely to have acute malnutrition when compared to their counterparts. This finding is in line with the study conducted in Shashogo Woreda. This might be due to the fact that taking the sick child earlier is advisable because it prevents further disease progress and complication and it also helps to detect the problem earlier.

This study showed that children from father who made decision alone on the child care and treatment were 4.78 times more likely to suffer from undernutrition and children from mother who made decision alone on the child care and treatment were 2.39 times more likely to suffer from acute malnutrition when compared to children from parents who made decision jointly. The study conducted in Haro Maya supports this finding which revealed that the odds of acute malnutrition is higher among children from parents who made decision individually [21]. This might be because women in low-income countries and those who have no education are often excluded from household decision-making, and this exclusion and power imbalance within relationship interfere with women's access to reproductive and child health service which in turn have adverse effect on child nutritional status.

According to this study, the odds of acute malnutrition is higher among children who stopped exclusive breastfeeding before the first six months of age and children of parents who discontinue breastfeeding before 24 months of birth. This finding is supported by the studies done in different parts of Ethiopia [21, 24, 26, 28]. This could be explained by the fact that breast milk provides many of the nutritional requirements of a child and contains anti-infective properties that protect children from early infections and enhance normal child growth. Besides, the longer duration of breastfeeding has a positive impact on linear growth of the child and prevention of chronic illnesses since it contains protective factors. Therefore, mothers who stop breastfeeding earlier might expose the children to malnutrition due to lack of important nutrients which increases the risk of infection.

The present study observed that the likelihood to be malnourished is higher among children who consume from less than four food groups in a day and children from households which have food insecurity problem. The study done in different geographic areas agreed with this finding [19, 20, 24, 33]. This is due to the fact that food insecure households might not satisfy their dietary needs and parents who have inadequate dietary diversity may expose children to infection due to poor immunity which in turn causes malnutrition.

## 5. Conclusion

This study identified various determinants of acute malnutrition and provided clues on the commonest contributing factors of acute malnutrition among under-five children in the study area. The major determinants of acute malnutrition among under-five children in this study were marital status, family size of five and above, birth interval of less than 2 years, mothers who had no formal education, birth weight less than 2.5 kg, diarrhea in the past two weeks before data collection, not taking sick children to health institutions within 24 hours, children from the household who use nonimproved toilet, children of parents who use water from unprotected sources, duration of exclusive breastfeeding, duration of breastfeeding for less than 24 months, inadequate dietary diversity, and household food insecurity. Thus, health education should be given to the community on child feeding practice, exclusive breastfeeding, and family planning. In addition, due attention should be given to growth monitoring and screening of the children at the clinical and community level to detect and tackle the problem at early stage. Besides, encouraging women's education and empowerment through awareness creating and capacity building are recommended.

## 6. Limitation of the Study

Since it was based on respondents' self-reported data and tracking of exposure status retrospectively, which was prone to recall bias, efforts were made to minimize it by giving detailed instructions for participants and providing adequate time for study participants to recall as much as possible.

## Figures and Tables

**Table 1:** Sociodemographic characteristics of study participants in public health facilities in Sodo town, 2021.

Variable	Categories	Cases	Controls	Total
Marital status	Married	100 (75%)	256 (96%)	356 (89.2%)
	Others	33 (24.8%)	10 (3.8%)	43 (10.8%)
Residence	Rural	55 (41.4%)	73 (27.4%)	128 (32%)
	Urban	78 (58.6%)	193 (72.6%)	271 (68%)
Religion	Protestant	76 (57.1%)	147 (55.3%)	213 (53.3%)
	Orthodox	49 (36.8%)	95 (35.7%)	144 (36%)
	Muslim	6 (4.5%)	24 (9%)	30 (8%)
	Other	2 (1.5%)	“—”	2
Mothers' education	No formal education	37 (27.8%)	14 (5.3%)	51 (12.8%)
	Primary	55 (41.4)	117 (44%)	172 (43.1%)
	Secondary and above	41 (30.8%)	135 (50.8%)	176 (44.1%)
Fathers' education	No formal education	15 (11.3%)	12 (4.5%)	27 (6.8%)
	Primary	55 (41.4%)	101 (38%)	156 (39%)
	Secondary and above	63 (47.4%)	153 (57.5%)	216 (54.1%)
Mothers' occupation	Trader	18 (13.5%)	39 (14.7%)	57 (14.3%)
	Employee	12 (9%)	85 (32%)	97 (24.3%)
	Daily laborer	29 (21.8%)	30 (11.3%)	59 (14.8%)
	Housewife	74 (55.6%)	112 (42.1%)	186 (46.6%)
Fathers' occupation	Trader	35 (26.3%)	72 (27.1%)	107 (26.8%)
	Employee	24 (18%)	115 (43.2%)	139 (34.8%)
	Daily laborer	56 (42.1%)	40 (15%)	96 (24%)
	Farmer	18 (13.5%)	39 (14.7%)	57 (14.3%)
Average monthly income (Ethiopian birr)	<1000	31 (23.3%)	9 (3.4%)	40 (10.1%)
	1001-1500	15 (11.3%)	14 (5.8%)	29 (7.3%)
	1501-2000	24 (18%)	21 (7.9%)	45 (11.3)
	2001-2500	5 (3.8%)	19 (7.1%)	24 (6.1%)
	2501-3000	14 (10.5%)	33 (12.4%)	47 (11.8%)
	>3000	44 (33.1%)	170 (63.9%)	214 (53.4%)

**Table 2:** Child characteristics, 2021.

Variable	Categories	Case	Control	Total
Sex	Male	76 (57.1%)	166 (62.4%)	242 (60.6%)
	Female	57 (42.9%)	100 (37.6%)	157 (39.4%)
Birth order	1-3	58 (43.6%)	169 (63.5%)	227 (56.9%)
	4 and above	75 (56.4%)	97 (36.5%)	172 (43.1%)
Child age (in month)	6-11	28 (21.1%)	52 (19.5%)	80 (20%)
	12-23	36 (27.1%)	74 (27.8%)	110 (27.6%)
	24-35	35 (26.3%)	70 (26.3%)	105 (26.3%)
	36-47	16 (12%)	44 (16.5%)	60 (15.1%)
	48-59	18 (13.5%)	26 (9.8%)	44 (11%)
Birth weight	<2.5 kg	18 (43.9%)	15 (15.5%)	33 (24%)
	≥2.5 kg	23 (56.1)	82 (84.5%)	105 (76%)

**Table 3:** Child caring and feeding practice of parents, 2021.

Variable	Categories	Case	Control	Total
Duration on exclusive breastfeeding	<6 months	111 (83.5%)	98 (36.8%)	209 (52.4%)
	>6 months	6 (4.5%)	14 (5.3%)	20 (5%)
	At six months	16 (12%)	154 (57.9%)	170 (42.6%)
Bottle feeding	Yes	89 (66.9%)	168 (63.2%)	257 (64.4%)
Give colostrum for the child	No	97 (72.9%)	112 (42.1%)	209 (52.4%)
Separate feeding plate	No	50 (37.6%)	113 (42.5%)	163 (40.9%)
Handwash before feeding the child	No	42 (31.6%)	54 (20.3%)	96 (24.1%)
Dietary diversity	Inadequate	103 (77.4%)	88 (33.1%)	191 (47.9%)
Household food insecurity	Food insecure	117 (88%)	124 (46.6%)	241 (60.4%)

**Table 4:** Determinants of acute malnutrition among under-five children in the study area, 2021.

Variable	Cases	Controls	COR (95% CI)	AOR (95% CI)	*P* value
Marital status					
Married	100 (75%)	256 (96%)	1	1	
Others	33 (24.8%)	10 (3.8%)	8.45 (4.01, 17.8)	4.45 (1.72, 11.5)	0.002^∗∗^
Residence					
Rural	55 (41.4%)	73 (27.4%)	1.86 (1.20, 2.88)	1.55 (0.70, 3.45)	0.273
Urban	78 (58.6%)	193 (72.6%)	1	1	
Family size					
1-4	79 (59.4%)	215 (80.8%)	1	1	
5 and above	54 (40.6%)	51 (19.2%)	2.88 (1.81, 4.57)	2.54 (1.37, 4.73)	0.003^∗∗^
Birth interval					
<24 months	59 (44.4%)	56 (21.1%)	2.99 (1.90, 4.69)	2.71 (1.54, 4.76)	0.001^∗∗^
≥24 months	74 (55.6%)	210 (78.9%)	1	1	
Mothers' education					
No formal education	37 (27.8%)	14 (5.2%)	8.70 (4.29, 17.65)	2.96 (1.02, 8.56)	0.044^∗^
Primary	55 (41.4)	117 (44%)	1.54 (0.96, 2.48)	0.73 (0.38, 1.39)	0.344
Secondary and above	41 (30.8%)	135 (50.8%)	1	1	
Mothers' occupation					
Trader	18 (13.5%)	39 (14.7%)	0.69 (0.37, 1.31)	1.29 (0.57, 2.94)	0.536
Employee	12 (9%)	85 (32%)	0.214 (0.10, 0.4)	0.47 (0.19, 1.17)	0.105
Daily laborer	29 (21.8%)	30 (11.3%)	1.46 (0.81, 2.63)	0.68 (0.28, 1.65)	0.399
Housewife	74 (55.6%)	112 (42.1%)	1	1	
Fathers' occupation					
Trader	35 (26.3%)	72 (27.1%)	1	1	
Employee	24 (18%)	115 (43.2%)	0.43 (0.236, 0.78)	0.66 (0.306, 1.45)	0.309
Daily laborer	56 (42.1%)	40 (15%)	2.88 (1.62, 5.1)	1.18 (0.50, 2.80)	0.699
Farmer	18 (13.5%)	39 (14.7%)	0.94 (0.47, 1.89)	0.16 (0.05, 0.50)	0.206
Birth order					
1-3	58 (43.6%)	169 (63.5%)	1	1	
4 and above	75 (56.4%)	97 (36.5%)	2.25 (1.47, 3.44)	2.61 (0.96, 7.09)	0.06
Birth weight					
<2.5 kg	18 (43.9%)	15 (15.5%)	4.27 (1.87, 9.77)	3.56 (1.15, 10.99)	0.027^∗^
≥2.5 kg	23 (56.1)	82 (84.5%)	1	1	
Diarrhea in the last two weeks					
No	58 (44.3%)	159 (76.8%)	1	1	
Yes	73 (55.7%)	48 (23.2%)	4.16 (2.60, 6.68)	9.15 (2.83, 29.53)	0.001^∗∗∗^
Pneumonia					
No	95 (73.1)	164 (79.2%)	1	1	
Yes	35 (26.9%)	43 (20.8%)	1.40 (0.84, 2.34)	3.05 (0.78, 11.85)	0.106
Antenatal care follow-up					
No	60 (45.1%)	75 (28.2%)	2.09 (1.35, 3.22)	1.06 (0.59, 1.90)	0.837
Yes	73 (54.9%)	191 (71.8%)	1	1	
Place of delivery					
Home	30 (22.6%)	42 (15.8%)	1.55 (0.92, 2.62)	0.54 (0.26, 1.12)	0.099
Health facility	103 (77.4%)	224 (84.2%)	1	1	
Postnatal care follow-up					
No	53 (39.8%)	84 (31.6%)	1.43 (0.93, 2.21)	0.94 (0.54, 1.65)	0.853
Yes	80 (60.2%)	182 (68.4)	1	1	
Immunization status					
Immunized	88 (66.2%)	205 (77.1%)	1	1	
Not immunized	45 (33.8%)	61 (22.9%)	1.71 (1.08, 2.72)	1.02 (0.54, 1.92)	0.952
Visit health facility within 24 hours of the onset of any sickness					
No	103 (81.1%)	105 (40.2%)	6.37 (3.83, 10.6)	5.10 (2.94, 8.86)	0.001^∗∗∗^
Yes	24 (18.9%)	156 (59.8%)	1	1	
Vaccinated for tetanus toxoid					
No	64 (48.1%)	93 (35%)	1.72 (1.13, 2.63)	0.88 (0.49, 1.58)	0.678
Yes	69 (51.9%)	173 (65%)	1	1	
Decision on health care of child					
Father only	54 (40.6%)	27 (10.2%)	6.95 (4.04, 11.96)	4.78 (2.52, 9.07)	0.001^∗∗∗^
Mother only	18 (13.5%)	27 (10.2%)	2.31 (1.19, 4.48)	2.39 (1.11, 5.16)	0.026^∗^
Jointly	61 (45.9%)	212 (79.6%)	1	1	
Type of toilet					
Improved	9 (6.8%)	104 (39.4%)	1	1	
Nonimproved	123 (93.2%)	160 (60.6%)	8.88 (4.32, 18.25)	5.09 (2.40, 10.79)	0.001^∗∗∗^
Drinking water					
Protected	76 (57.1%)	236 (88.7%)	1	1	
Nonprotected	57 (42.9%)	30 (11.3%)	5.9 (3.53, 9.84)	3.45 (2.00, 5.93)	0.001^∗∗∗^
Duration of exclusive breastfeeding					
<6 months	111 (83.5%)	98 (36.8%)	10.90 (6.09, 19.51)	4.65 (2.21, 9.78)	0.001^∗∗∗^
>6 months	6 (4.5%)	14 (5.3%)	1.39 (12.22)	4.46 (1.02, 19.45)	0.046^∗^
At six months	16 (12%)	154 (57.9%)	1	1	
Give colostrum for child					
No	97 (72.9%)	112 (42.1%)	3.7 (2.35, 5.83)	1.40 (0.73, 2.68)	0.297
Yes	36 (27.1%)	154 (57.9%)	1	1	
Counseling on child feeding					
No	62 (46.6%)	78 (29.3%)	2.1 (1.36, 3.23)	0.60 (0.32, 1.13)	0.116
Yes	71 (53.4%)	188 (70.7%)	1	1	
Duration of breastfeeding					
<24 months	124 (93.2%)	97 (36.5%)	24 (11.67, 49.37)	10.26 (4.57, 22.99)	0.001^∗∗∗^
≥24 months	9 (6.8%)	169 (63.5%)	1	1	
Dietary diversity					
Inadequate	103 (77.4%)	88 (33.1%)	6.94 (4.29, 11.22)	2.05 (1.05, 4.00)	0.034^∗^
Adequate	30 (22.6%)	178 (66.9%)	1	1	
Food insecurity					
Food secure	16 (12%)	142 (53.4%)	1	1	
Food insecure	117 (88%)	124 (46.6%)	8.37 (4.71, 14.88)	3.83 (1.80, 8.14)	0.001^∗∗∗^

Statistically significant at ^∗^*P* value < 0.05, ^∗∗^*P* value < 0.01, and ^∗∗∗^*P* value < 0.001. 1 = reference category; COR = crude odds ratio; AOR = adjusted odds ratio; CI = confidence interval.

## Data Availability

The data set used in this study will be available from the corresponding author at a reasonable request.

## Abbreviations

ANC: Antenatal care
AOR: Adjusted odds ratio
CI: Confidence interval
EBF: Exclusive beast feeding
EDHS: Ethiopian Demographic and Health Surveillance
HIV/AIDS: Human immunodeficiency virus/acquired immunodeficiency syndrome
MAM: Moderate acute malnutrition
MCH: Maternal and child health
MUAC: Middle upper arm circumference
PNC: Postnatal follow-up
SAM: Severe acute malnutrition
UNICEF: United Nations Children's Fund
WHO: World Health Organization.
